# Developing a cognitive assessment toolkit for primary care: qualitative assessment of providers’ needs and perceptions of usability in clinical practice

**DOI:** 10.1186/s12913-023-09991-7

**Published:** 2023-09-19

**Authors:** Monica Zigman Suchsland, Barak Gaster, Jaqueline Raetz, Basia Belza, Lisa McGuire, Benjamin Olivari, Karen Tracy, Annette L. Fitzpatrick

**Affiliations:** 1 Department of Family Medicine, University of Washington, 1959 NE Pacific St, Box 356390, Seattle, WA 98195-6390 USA; 2 Department of Medicine, University of Washington, 1959 NE Pacific St, Box 356420, Seattle, WA 98195-6390 USA; 3 School of Nursing, University of Washington, 1959 NE Pacific St, Box 357260, Seattle, WA 98195- 6390 USA; 4 National Center for Chronic Disease Prevention and Health Promotion, Centers for Disease Control and Prevention, 1600 Clifton Road, Atlanta, GA 30329-4027 USA; 5 The Gerontological Society of America, 1220 L Street NW, Suite 901, Washington, DC 20005 USA; 6 Departments of Epidemiology and Global Health, University of Washington, UW Box 351619, Seattle, WA 98195 USA

**Keywords:** Dementia, Cognition, Memory, Aging, Assessment, Evaluation, Diagnosis, Referral

## Abstract

**Background:**

Burden of dementia is expected to substantially increase. Early dementia is underdiagnosed in primary care. Given the benefits of active management of dementia, earlier detection in primary care is imperative. The aim of this study was to understand primary care provider (PCP) perceptions of implementing a cognitive assessment toolkit in primary care.

**Methods:**

PCPs in a large health system in the US were recruited to a qualitative study utilizing semi-structured interviews. Interviews captured provider perceptions of options for implementing a cognitive assessment toolkit derived from the Gerontological Society of America (GSA) KAER (Kickstart, Assess, Evaluate, Refer) toolkit, including a workflow and adapted clinical tools. A content analysis approach distinguished themes and exemplary quotes.

**Results:**

Ten PCPs were interviewed. They found the toolkit useful, felt the term Kickstart was not specific to dementia care, and stressed that addressing cognitive evaluation would need to be easy to implement in a clinical workflow. Finally, providers knew many resources for referral but were unsure how to help patients navigate options.

**Conclusions:**

Providers stressed simplicity, ease, and efficiency for implementation of a cognitive assessment toolkit. Incorporating these findings into the development of clinical tools and workflows may increase cognitive evaluations conducted by PCPs.

## Background

It is estimated that the global burden of dementia is going to triple in the next 30 years, from a current prevalence of 57 million cases to over 150 million cases [1]. The growing older adult population and the rise of other chronic diseases are main contributors to this projected increase [1]. The current burden in the US is large as well and is expected to double by 2060 [2]. Between 2019 and 2020, it was estimated that deaths in the US from Alzheimer’s disease increased 145% [3]. Along with increasing prevalence, there will be a substantial increase in societal burden from this disease. It is estimated that Alzheimer’s and other dementias will cost the US $355 billion [4]. Furthermore, it is estimated that a person with dementia lives with disability for an average of 11.9 years, and this figure is expected to increase with lengthening life expectancy [5]. Complicating the burden is the fact that dementia is likely under-diagnosed and under-treated. It is estimated that 50% of early cases are missed in primary care [6]. Factors contributing to the under diagnosis and treatment of dementia by primary care providers (PCPs) include patient hesitancy due to the stigma of the disease, under-recognizing symptoms, demands on provider time, and lack of confidence in diagnosing and treating dementia among PCPs [6–8].

The expanding prevalence of dementia, along with its under diagnosis, has motivated a national effort to create better guidance for early diagnosis in primary care [8, 9]. Recognition and treatment of dementia falls well within the scope of primary care given the definition of primary care as “the provision of integrated, accessible health care services by clinicians who are accountable for addressing a majority of personal health care needs, developing a sustained partnership with patients, and practicing in the context of family and community.” [10]. There is ample evidence that certain modifiable risk factors are associated with the incidence of dementia and that dementia may be preventable [8, 11, 12]. Early diagnosis also allows people with dementia to plan for the future, find support, participate in clinical trials, and receive treatment early, [8] thereby improving quality of life. The Gerontological Society of America (GSA) developed the GSA KAER toolkit for PCPs to improve awareness, detection, and referral for services for patients with dementia [9].

The GSA KAER toolkit has four steps: (1) **K**ickstart the brain health conversation by observing and listening for patient signs or concerns about cognition; (2) **A**ssess the need for a full cognitive evaluation; (3) **E**valuate for cognitive impairment; and (4) **R**efer the patient and family for community services and resources [9]. This toolkit provides a comprehensive list of techniques and guidance to improve detection of mild cognitive impairment and dementia in the primary care settings and was designed to provide wide flexibility to meet the needs of individual healthcare systems. However, the toolkit has yet to be widely used in primary care. As part of a larger project that plans to review, refine, pilot test, and evaluate the GSA KAER toolkit, we sought to understand PCPs perceptions of adapting the GSA KAER model in primary care settings.

## Methods

A qualitative study of semi-structured interviews was conducted. PCPs (nurse practitioners, physicians, and physician assistants) were recruited from a 16-clinic primary care network in a large health system in Washington State. Participants were asked to volunteer through an email sent to a PCP listserv within the health system. A convenience sample of volunteer participants was enrolled. The University of Washington Human Subjects Division reviewed all experimental protocols and approved all protocols for this project under the designation of quality improvement and not human subjects research (IRB #: STUDY00012214). Informed verbal consent was provided by each interviewee since interviews were conducted remotely over a video conferencing platform, the University of Washington Human Subject Division approved the verbal consent method. All methods were carried out in accordance with relevant guidelines and regulations outlined by the University of Washington Human Subjects Division. Participants received a $100 gift card as compensation for their time.

The GSA KAER model was developed with a focus on 4 steps to guide primary care clinicians on managing cognitive health among their patients. Each step in the model (Kickstart, Assess, Evaluate, and Refer) has specific approaches to addressing the topic in clinical practice (21 total approaches) and various tools to aid each step. The intended purpose of the model is to adapt it to fit the needs of specific health systems. For example, the model provides 7 possible approaches for the Kickstart step (“Raise the topic of brain health; Ask about memory and cognition; Listen for older adults’ concerns about memory and cognition; Listen for family concerns about the older adult’s memory and cognition; Observe for signs and symptoms of cognitive impairment; Add a question about memory or cognition to health risk questionnaires; Use information about health conditions and functioning from existing patient records”) and suggests combining two or more of them [9].

To develop a clinic workflow utilizing a variety of tools recommended by the GSA KAER model, an interview guide was created to describe to clinicians how the GSA KAER model and clinic tools could be implemented and utilized in primary care. The interview guide asked for provider preferences and workflow suggestions when multiple tools were presented. From the *Kickstart* section we discussed brain health conversations and triggers to warrant taking further clinical action to assess cognition. Three potential assessment tools were presented from the *Assess* step. We asked for feedback on draft electronic health record tools to aid the evaluation visit.

Interviewers (BG and JR) were two primary care physicians, one male and one female, with expertise in gerontology. The two interviewers both work in the same health system as PCPs invited for interview but do not work at the same clinic location as each other or with any of the PCPs that participated in an interview. Therefore, some interviewees were work acquaintances with the interviewers, but interviewees were not direct colleagues. The goal of the interview was outlined in the interview guide and described to interviewees. Sessions were led by both interviewers and conducted with either one or two PCPs at a time. Interviews were conducted during April and May 2021. Each interview lasted 45–60 min. Participants were enrolled and interviews were conducted until data saturation was reached [13, 14]. Interviews were done exclusively over video conferencing, audio and visually recorded, and transcribed. Video conferencing was selected to abide by COVID-19 safety regulations and to provide flexibility for timing to maximize participation. Interviewers shared field notes with the project team after the interviews.

Data were organized and analyzed using the Microsoft Word comment feature to highlight quotes pertaining to specific codes. Coded versions of transcripts were merged into one document and highlighted quotes were extracted into a table. A deductive content analytic approach informed the analytic process [15]. Initial codes were predetermined from the GSA KAER model: Kickstart, Assess, Evaluate, Refer. After immersion by three coders (BB, AF, MZS) in 2 transcripts, new codes appeared and were included in the coding framework: time, barriers, clinical presentation, tools, who can help, pandemic, resource suggestions, provider perceptions, and provider awareness. The remaining transcripts were coded by two of the three coders and reconciled by the third coder. Excerpts/quotes were organized by code and compared across interviews. Themes emerged and were organized within the GSA KAER model categories. Participants did not review transcripts or provide feedback on the final themes. This report is in adherence with COREQ guidance [16].

## Results

Ten PCPs completed interviews, 3 interviews were conducted with 2 PCPs at once, and 4 interviews were conducted with 1 PCP. Of those, 9 were female and 1 was male; 9 were physicians and 1 a nurse practitioner. Clinical experience ranged from early (4.5 years) to late career (28 years). Themes and supporting quotes were grouped by current practice of cognitive evaluation in primary care and the GSA KAER model terms, *Kickstart, Assess, Evaluate, Refer*.

### Current practice

Study participants did not report a clear method for determining if a cognitive evaluation was needed (Table 1). A variety of methods were described, including, reviewing care history for repeated calls for the same indication or missed visits, patient/family member initiation, or provider noticed changes. When cognitive assessments were completed, providers described the process either as routinely done or “hit or miss”. The type of assessment tool varied according to provider preference. Provider time, patient reluctance or anxiety, and provider hesitation were the most reported barriers to conducting cognitive assessments during a primary care visit. Providers conveyed concern about what to do with a positive finding, described difficulty picking up on subtle changes in cognition, and felt they lacked understanding of what to do with specific assessment results.

**Table 1:** Practice Themes as Identified by Participants

Theme	Examples from Study Participants
Deciding which patient would benefit from a cognitive evaluation isn’t always clear.	I’m sure there’s room for improvement. I think I usually assess for cognitive function when a patient brings up a concern. I don’t think I am usually the one to initiate that process. – I02
Consistently assessing patients is challenging.	I’d say it’s rather hit or miss. I’d say probably I do it most consistently if I’m doing a Medicare Annual Wellness Visit. Otherwise, it’s based on a patient concern or family member concern. – I07 If you have rapport with patients and you’ve been seeing them for a long time it’s hard to say let’s assess you cognitively. They don’t want to be assessed because they might have some suspicion that there’s some decline, and they just don’t want to be assessed. It is helpful to have the Medicare Wellness Visit where we do a cognitive assessment or screening. – I05
There are barriers to performing cognitive evaluations.	I would need to know how to better do an assessment specifically around mild cognitive impairment. – I01 In primary care where there’s 1,000 other things to address this is just one element of many. You’re hoping no one says they have concerns for memory. – I03 I don’t like to bring up memory issues, especially when I have no answers for them and it can just cause anxiety. – I01 I don’t have a good framework for how to counsel people on their diagnosis. – I02

### Kickstart

Provider appreciation of the term *Kickstart* in reference to cognitive health varied (Table 2). Some expressed that the term made them think of a “fundraiser” or an “exercise program” instead of the intended meaning of being on the look-out for warning signs of cognitive impairment. When the goal of the term *Kickstart* was explained, PCPs identified other terms that resonated with them more such as “eyes open”, “be aware”, “radar” or “have your antenna up.” An approach suggested for the *Kickstart* step is to discuss brain health as part of dementia prevention, however, one provider felt this discussion belonged with assessment and counseling to motivate patients to take steps for dementia prevention such as drinking less alcohol.

**Table 2:** Themes within the GSA KAER Model and Exemplary Quotes

Theme	Examples from Study Providers
The term Kickstart elicits a number of interpretations.	The kickstart is not specific to cognitive health, so it doesn’t resonate with me in terms of cognitive impairment and dementia evaluation. – I04 If you change the Kickstart, then you wouldn’t have that acronym as well… That’s a nice feature of it. I don’t have any real feeling pro or con Kickstart. I think what you want, is something that in the middle of a busy day when you haven’t thought about it, maybe in a while you’re like oh what’s that acronym again and, what is the K. That’s sort of the process that happens is because it’ll come up…sporadically and you want something that you can remember. In that sense, I think the K, fits with the overall acronym. – I07 I need to be intentional about paying attention if a patient expresses concerns or a family member has expressed concerns about their memory. If this is a patient who has recurrent missed appointments. It wasn’t their intention to miss it, but perhaps they forgot about it, to bring more awareness. Being aware, more attention grabbing than kickstart. My patients with dementia or mild cognitive impairment fake it well. – I03
Providers desire simplicity and clear recommendations regarding assessment.	What’s the most effective way of figuring out if someone has mild cognitive impairment and then add that to what we’re doing if we’re missing that… integrate in a way that doesn’t add a new form – I07 The Mini-Cog is limiting, but it is what I do the most… With my own practice of patients with dementia and the Mini-Cog in and of itself is very limiting just from a pragmatic perspective, even though it’s evidence based it’s pretty limiting. I have to have the threshold myself to say you know what I still think you need to have a MoCA or I still think we need to advance the conversation. - I03
EMR tools for evaluation should be efficient and streamlined.	[smart sets] I have some that some seem helpful to me, and there are others out there that do not and the ones that I don’t like are the ones that… just consider too busy, too many things. – I05 The dot phrase [workflow tool] actually can be super helpful when they’re not a full note because you can have all of these really concise lists like this of just check boxes to just remind yourself of what you need do. – I01 I like having a structured list of normal signs of aging… that would be a really useful tool if it covers a lot of the questions I’m asking on my own, and then it gives patients some structure and you can potentially follow that over time if it’s a continual concern. – I06
Referral to community resources is difficult.	I give a ton of verbal information, but if you have memory impairment you’re not going to hold on to 90% of that probably, and so I try to put everything in writing in my after visit summary. – I04 Our social worker seems really busy doing counseling and I’m not sure if she knows resources in the community for dementia, I know that our psychiatrist doesn’t. – I06 I think care provider respite is something that people have had problems with, more support groups that are more varied and maybe culturally appropriate, more expectant guidance for care providers and patients on the progression of their MCI or their dementia, I think there’s so much fear. And I still don’t totally understand how people get like home health aides for like chores, the logistics of that as well as the actual services. Resources on like driving assessment, an alternative for seniors who are no longer able to drive or shouldn’t be driving… what are you going to do if you can’t… it really takes away people’s freedom. – I02

### Assess

Providers valued a structured and systematic approach to implementation of the assessment step. Providers approved of short and simple assessments such as an abbreviated version of the *Alzheimer’s Association 10 Warning Signs* [17]. They heavily stressed that if the step added more work, they were unlikely to adopt it. Provider descriptions for the Mini-Cog screening test varied to include descriptions such as cumbersome, easy, “takes too much time”, a good gauge of cognitive impairment, and even if it was normal a full cognitive evaluation may still be needed. Assessments during a typical clinic visit should be easy to do and must be able to be integrated in the time already allotted for the visit, such as while the patient is waiting (Table 2).

### Evaluate

Providers discussed the utility of tools during an evaluation for dementia. The length of the tool and the time it takes to use it was determined by some providers to impact whether the tool would be used in a patient encounter (Table 2). Providers felt a checklist to guide cognitive evaluations would be helpful and prevent them from missing key components during the evaluation. They expressed organization and efficiency of the tool as important. Similar to the *Assess* step, they voiced concern that tools that were not well designed or took too much time would not be used.

### Refer

Providers were aware of a large number of community resources. Despite knowledge of many different resources, providers were uncertain what resources would be most helpful for their patients with a new diagnosis of dementia. They did not point to one primary resource that they rely on. Providers expressed concern about availability or accessibility of referring to specialty care, support services such as a social worker, or outside resources (Table 2). Providers who had fully staffed clinics with either a social worker or health navigator relied on them to refer patients to community resources. However, providers didn’t utilize the clinic social worker or navigator to refer resources when clinics were stretched thin or providers felt the clinic social worker or health navigator’s area of expertise was not geriatrics/dementia care.

## Discussion

Implementation of the GSA KAER toolkit in primary care is a promising method to promote earlier diagnosis of dementia and dementia prevention [9]. This is the first study examining PCPs’ perceptions of this model. To help plan the adaptation of the GSA KAER toolkit to a large health system, PCPs reflected on how the toolkit would be used in primary care practice. Providers stressed workflow simplicity, ease, efficiency, and specifically outlined steps as imperative to implementing the GSA KAER toolkit. The *Assess* step in particular, deciding when to proceed to a full *Evaluation* step, cannot create more work or undue burden for the provider.

Two studies present similar challenges and opportunities in diagnosis of cognitive impairment in primary care. One program implemented an electronic medical record clinical tool, such as those that would be used in the *Evaluation* step of the KAER model [18]. The tool was found to increase provider confidence in diagnosis but did not increase rates of diagnosis. A second study found positive associations with provider training aimed to improve diagnosis and treatment of people with dementia and increased knowledge about it [19]. Given the small sample sizes and scope of previous research (including this current study), more research is clearly needed to increase understanding of implementation of programs aimed at improving early assessment and diagnosis of dementia.

This project is not without limitations. Interviewers and interviewees were colleagues within the same health system, potentially introducing social desirability bias. To overcome this possibility, the interview guide was developed to ask open-ended questions and interviewees were encouraged to provide their perspective. In addition, the results may have been subject to confirmation bias by the point of view of the researchers. To address this, quotes to support and weaken themes were collated comprehensively and considered fully when finalizing the analysis. The study sample is limited to a convenience sample within a single health system, mainly female and trained as physicians. As a result of the convenience sample, those that volunteered to participate may have had a higher interest in dementia assessment. Although this may impact generalizability, the themes identified in this study were broad. Future research should confirm these findings in broader primary care populations.

## Conclusions

This is the first published study to investigate factors that would influence the success of the GSA KAER model implemented in primary care. Main findings from this study identify specific methods that can improve acceptance and usability of the model. This study will inform implementation of the GSA KAER toolkit in primary care clinics across a large health system. Specific changes to be made to the toolkit for implementation in the health system include a rebranding of *Kickstart* to *Be Aware*, shortening a longer assessment tool to fit into a typical appointment timeslot, and electronic health record tools developed to simplify the evaluation and counseling step. Additionally, all tool adjustments and resources will be made available on the project website (cognition-primarycare.org). Incorporating these findings into the development of clinical tools and workflows derived from the GSA KAER model may increase the model’s usage among PCPs, thereby improving early assessment and diagnosis of dementia. If successfully implemented, next steps in research will be to determine if implementation of the model increases early assessment and diagnosis.

## Data Availability

Available upon request to the corresponding author.

## Abbreviations

GSA: Gerontological Society of America
IRB: Institutional Review Board
PCP: Primary Care Provider
